# Clinical Management of Hydrops Fetalis in a Premature Neonate in India: A Case Report

**DOI:** 10.7759/cureus.64464

**Published:** 2024-07-13

**Authors:** Mridu Bahal, Sudhir Malwade, Jasleen Dua, Abhishek Denge, Sheuli Paul

**Affiliations:** 1 Pediatrics, Dr. D. Y. Patil Medical College, Hospital & Research Centre, Pune, IND; 2 Pediatric Medicine, Dr. D. Y. Patil Medical College, Hospital & Research Centre, Pune, IND

**Keywords:** pediatrics & neonatology, blood group rh(d)-negativity, coombs positive hemolysis, rhesus incompatibility, minor blood group incompatibility, hydrops fetalis

## Abstract

Hydrops fetalis has classically been defined as the presence of extracellular fluid in at least two fetal body compartments. This fluid collection includes skin edema (> 5 mm thickness), pericardial effusion, pleural effusion, and ascites. Here we present a case of a 29-year-old female with antenatally diagnosed severe hydrops fetalis which was postnatally successfully managed. Despite recent advances, immune hydrops are still a challenge for healthcare workers in third-world nations.

## Introduction

Premature infants with immune hydrops fetalis (IHF) face heightened risks due to their underdeveloped organ systems and complications associated with prematurity, such as respiratory distress, underdeveloped skin barriers, and immature cardiovascular and renal functions [1]. Managing IHF in these infants requires a multifaceted approach that addresses both the underlying immunological conflict and complications from premature birth. The pathophysiology of IHF in premature infants involves a complex interplay between maternal-fetal immunology and fetal development [2,3]. Maternal antibodies produced in response to previous sensitization events, such as prior pregnancies or blood transfusions, cross the placenta and attack fetal red blood cells (RBCs) [3]. This hemolysis leads to severe anemia, triggering compensatory mechanisms like increased erythropoiesis in the liver and spleen, ultimately resulting in hepatosplenomegaly and generalized fluid retention [4]. The prevalence of IHF has decreased in developed countries due to effective Rhesus (Rh) immunoglobulin prophylaxis [5]. However, it remains a significant concern in regions with limited access to such preventive measures. Risk factors include maternal Rh-negative blood type, previous sensitization events, and the presence of other blood group incompatibilities [1].

## Case presentation

A 29-year-old woman presented at 31 weeks’ pregnancy, G3P2L1D1, with a positive indirect Coombs test. Ultrasonography suggested a single viable fetus with polyhydramnios, placentomegaly, significant ascites, cardiomegaly, and subcutaneous edema. The mother had no history of fever with rash, drug intake, or uterine bleeding and received dexamethasone 24 hours prior to delivery. She had a history of intrauterine death at eight months of gestation and received anti-D in both pregnancies.

**Figure 1:**
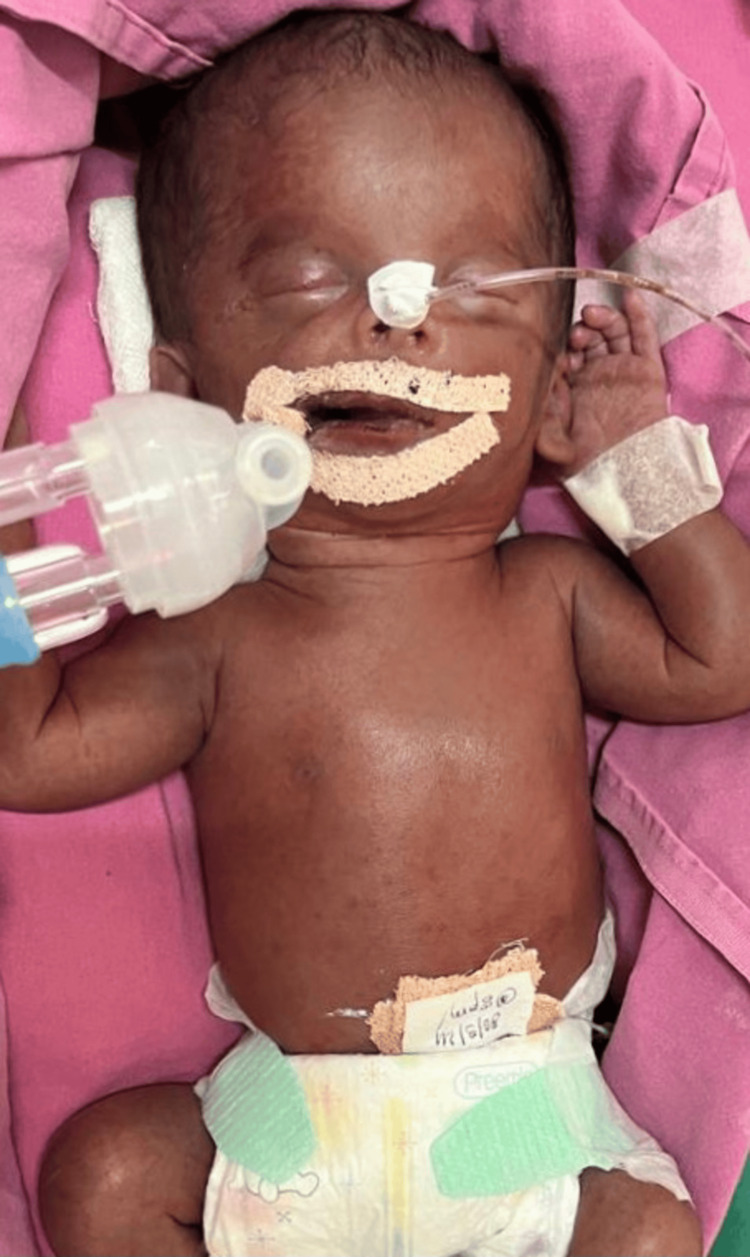
Neonate with Hydrops Fetalis

A male baby was born via lower segment cesarean section in view of fetal hydrops and polyhydramnios, with a birth weight of 1.9 kg (Figure 1). He was intubated and mechanically ventilated. The baby received surfactant at birth, a line was secured, and he was kept nil by mouth and managed with inotropes due to systemic hypotension. Initial labs showed that serum bilirubin was above the exchange cutoff with profound anemia (Table 1).

**Table 1:** Blood Parameters

Hour of Life	Birth	20 Hours	36 Hours	54 Hours	Reference Range
Hemoglobin	8.7	15.2	18.9	20	12.5 - 21 g/dL
Total Leucocyte Count	17600	10500	4000	3400	5000 – 21000 /µL
Platelet Count	1.82L	1.24	54000	35000	150000 – 21000 /µL
Packed Cell Volume	28.5	46	52.7	59.6	39 – 60%
Serum Albumin	2.5		2.5		3.5 - 5.2 g/dL
Total Bilirubin	12.32	14.3	8.5	4.19	-
Direct Bilirubin	0.45	0.79	0.8	0.76	-
Indirect Bilirubin	11.9	13.64	7.4	3.43	-
Phototherapy Cutoff	4	7	8 to 10	8 to 10	-
Exchange Cutoff	11.5	11.5	13-16	13-16	-

Intravenous immunoglobulin (IVIG) was administered while awaiting blood procurement. He received a double-volume exchange transfusion with O negative blood, intensive phototherapy, and continued IVIG. Procuring compatible blood was challenging due to 60-70% blood incompatibility on cross-matching. Eventually, a blood bag with 5-10% blood incompatibility was used for the double-volume exchange transfusion.

Subsequent labs done at 20 hours of life showed rising serum bilirubin and falling hemoglobin, necessitating a second exchange transfusion. IVIG was continued to decrease hemolysis. Following the second exchange transfusion, his ascites and pericardial effusion resolved, serum bilirubin decreased, and albumin and hemoglobin stabilized. Problems associated with prematurity persisted, as the baby failed an extubation trial and required DART (Dexamethasone: A Randomised Control Trial) and HCT (Hydrochlorothiazide) regimens twice. After completing the regimen, he was extubated and put on nasal CPAP (continuous positive airway pressure). His course was complicated by fungal sepsis, feeding intolerance, and prolonged oxygen dependence.

The infant was discharged from the NICU at 12 weeks of life (43 weeks corrected gestational age), with follow-up appointments scheduled for developmental assessment and monitoring for potential long-term complications.

## Discussion

The incidence of hydrops fetalis is approximately 1 in 1500 to 3800 births, with a perinatal mortality rate of around 60% [2]. In the first trimester, about 67% of pregnancies result in miscarriage or fetal death in utero (FDIU) [6]. During the second trimester, approximately 50% of pregnancies end in FDIU, and among those that progress to live births, there is a 20% neonatal mortality rate [7].

IHF primarily results from alloimmune hemolytic disease, where maternal antibodies cross the placenta and attack fetal RBCs, causing hemolysis and anemia [8]. This immune response is typically triggered by incompatibility between maternal and fetal blood group antigens, most commonly the Rh D antigen. Other antigens, such as Kell, Duffy, and Kidd, can also be involved [3,8]. The destruction of fetal RBCs leads to severe anemia, resulting in high-output cardiac failure. The heart's inability to maintain adequate circulation causes hydrops, characterized by generalized edema and effusions. Anemia activates compensatory mechanisms like increased erythropoiesis in the liver and spleen, leading to hepatosplenomegaly and further contributing to fluid imbalance [8]. The most common cause of IHF is Rh incompatibility, which occurs when a Rh-negative mother becomes sensitized to Rh-positive fetal RBCs. Sensitization typically occurs during a previous pregnancy, miscarriage, or blood transfusion. Subsequent pregnancies with a Rh-positive fetus lead to maternal anti-D antibodies attacking fetal RBCs. Although Rh incompatibility is the most well-known cause, its incidence has decreased significantly due to the widespread use of Rh immunoglobulin prophylaxis [5]. Nonetheless, other blood group incompatibilities and rare causes, such as maternal autoantibodies or fetal genetic disorders, continue to pose challenges. Enhanced imaging techniques and a better understanding of fetal monitoring have improved diagnostic accuracy and treatment outcomes. Despite progress, managing non-Rh alloimmunization remains challenging due to variability in antibody responses and a limited understanding of rare blood group antigens. Access to specialized care for intrauterine transfusion (IUT) and management of hydrops in low-resource settings also present significant challenges.

A rare case of immune hydrops was reported in India by Biswas et al. in a 23-year-old female (G2P1L1) with a history of double volume exchange transfusion in her first child [9]. The mother had blood group A RhD+ with an ICT titer of 1:128. Maternal antibodies showed agglutination on 3 and 11 cell panels with a negative auto control, and the suspected antibody was anti-Rh17. A proactive search for a compatible donor for intrauterine exchange transfusion was conducted, but multiple O and RhD-negative donors were incompatible with the mother's blood. Eventually, the patient's brother was found to be compatible. An ultrasound at 23 weeks gestation showed fluid in all body cavities of the fetus. Cordocentesis revealed the baby's hemoglobin was 2.8 g/dL, and the baby had a positive direct Coombs test (DCT) with blood group B+. Adsorption elution studies on red blood cells from cord blood showed pan-agglutination on 3 and 11 cell panels with a negative auto control. The fetus's blood group was ABO incompatible with the probable donor, so compatible blood for IUT could not be found, and the patient experienced an intrauterine fetal demise at 27 weeks gestation.

In another case reported by Tara et al., a 34-year-old (G4L2D1) with a positive ICT titer of 1:128 at 17 weeks gestation underwent intrauterine therapeutic plasma exchange [10]. Subsequent ultrasound examinations were normal, and at 37 weeks, the patient delivered a healthy male neonate weighing 2.520 kg, blood group O positive, DCT negative. The newborn was stable and discharged.

A similar case reported by Gurung et al. in 2023 involved a mother with a history of 14 pregnancies who did not receive anti-D immunoglobulin in her previous pregnancy [11]. An ultrasound revealed a single living fetus at 32 weeks gestation with significant fetal pericardial and pleural effusion, generalized body edema, enlarged subcutaneous tissue (>1 cm), placentomegaly, and polyhydramnios. The mother had a history of eight neonatal deaths, three intrauterine deaths, and two abortions, with an ICT titer of 1:69. A male infant born at 30 weeks, three days with generalized body edema required resuscitation at birth due to minimal breathing efforts and had refractory hypotension. Pericardiocentesis and ascitic tapping were performed, but the patient went into bradycardia, and despite CPR, the patient could not be revived.

In our study, the patient had similar ultrasound findings, was DCT positive, and required two exchange transfusions. Finding cross-matched blood was challenging, but eventually, an O-negative blood bag with 5%-10% blood incompatibility was found, which was used to manage the patient. Hyaline membrane disease complicated the case. Additionally, the patient developed chronic lung disease and was difficult to wean off oxygen. Despite these challenges, our case had a better prognosis compared to the other case reports.

## Conclusions

IHF remains a complex condition requiring multidisciplinary management. Early diagnosis through advanced imaging and laboratory tests, coupled with timely interventions like intrauterine transfusions, has improved outcomes. However, continuous research and improved access to specialized care are essential to further enhance prognosis and manage rare causes of IHF effectively.
